# Concise Update on Genomics of COVID-19: Approach to Its latest Mutations, Escalated Contagiousness, and Vaccine Resistance

**DOI:** 10.1055/s-0041-1725143

**Published:** 2021-03-15

**Authors:** Amir Khodavirdipour, Sarvin Jabbari, Fariba Keramat, Mohammad Y. Alikhani

**Affiliations:** 1 Department of Biology, Faculty of Natural Sciences, University of Tabriz, Tabriz, Iran; 2 Department of Anatomy, Division of Human Genetics, St. John's National Academy of Health Sciences, Bangalore, India; 3 Department of Infectious Diseases, Faculty of Medicine, Hamadan University of Medical Sciences, Hamadan, Iran; 4 Department of Microbiology, Hamadan University of Medical Sciences, Hamadan, Iran

**Keywords:** coronavirus, COVID-19, pandemic, viral outbreak, genomics of virus

## Abstract

The novel coronavirus disease 2019 (COVID-19) that started to invade the world from the Chinese fish market, causes an acute respiratory distress syndrome. COVID-19 is a dreadful infectious disease that surfaced only less than 8 months ago and caused the deadly COVID-19 pandemic. In this new species with a positive, single-strand RNA genome and a huge size, from the proteomics point view, there are no changes in sequences of amino acids in NSP7, 13, matrix, or envelope or other proteins including 8b and p6 and excluding NSP2 and NSP3. P6 is a multifunctional golgi–endoplasmic reticulum membrane-associated protein. This complex has a key duty to increase the replication rate of the virus and also causes intrinsic immune system responses by suppressing the signal transducer and activator of transcription factor 1 (STAT 1) translocated to the nucleus. Palmitoylated proteins elevate hydrophobicity which helps in membrane connection. Inside the N-linked glycosylation, moieties oligosaccharide is adhering to Asn-X-Ser/Thr canonical sequence. This helps for exact enfolding and carrying viral proteins by industriously using host's chaperon proteins including calreticulin and calnexin. 2B proteins encourage the internalization of major histocompatibility complex, class-I (MHC-I) protein and meanwhile inhibit their transfer to the surface of the cell as a recognition side. The deubiquitination of severe acute respiratory syndrome-coronavirus-2 (SARS-CoV-2) has precise modification apparatus in the posttranslational stage. In this article, we outlined the recent and up-to-date data on genomic and molecular structures, epidemiology, vaccine development, and, last but not least, the clinical features, diagnostics, and treatment of the novel coronavirus.

## Introduction

The severe acute respiratory syndrome-coronavirus-2 (SARS-CoV-2) from the family and subfamily of coronaviridae and coronavirinae, respectively, is a type of viruses with a positive single-strand RNA genome. 1 SARS-CoV-2 has the most enormous genome size (nearly 30 kb) among all RNA viruses. Based on genomics criteria, coronaviruses have been categorized into three subdivisions: α, β, and γ CoVs 2 ( Table 1 ). As of September 6, 2020, report, the number of confirmed cases crossed 27,000,000 positive cases and 890,000 deaths worldwide. 3 At first, China reported that pregnant ladies and children have rarely been affected with no mortality, but on March 24, 2020, Iran had been reported the death of two kids, 3 and 6 years old, in North Khorasan province 4 for the very first time in Iran and as of now, only in Isfahan city, “considerable” number of infants under the age of 1 to 2 months died due to COVID-19; Dr. Nasser Mostafavi from Isfahan University of Medical Sciences failed to pinpoint the exact number. 5 By the first week of August 2020, only a single hospital in Mashhad City of Khorasan Razavi province reported 370 positive cases in children along with 80 positive cases among the staff of the same hospitals including doctors and nurses. 6 The future of life and its condition with this virus is unclear. This review tried to give a bird's eye view on the new COVID-19. Our understanding regarding COVID-19 is less but growing momentarily and researchers are advised to keep them updated daily. COVID-19 is an enveloped positive single-strand RNA virus which has a key role in respiratory and enteric infections in animals and humans, with a diameter of 60 to 140 nm on the outer surface they have spike structures that can be seen under the electron microscope. There are four different types of coronavirus HKU1, OC43, 229E, and NL63 which previously reported in human and cause mild respiratory infections. 1

**Table 1:** Organization of coronavirus (CoV) species

Organization of CoV species (adapted from Jimenez-Guardeño et al) 2	
Group	Species
α-CoVs	Transmissible gastroenteritis coronavirus (TGEV) Canine coronavirus (CCoV) Porcine respiratory coronavirus (PRCoV) Feline coronavirus (FeCoV) Porcine epidemic diarrhea coronavirus (PEDV) Human coronavirus 229E (HCoV-229E) Human coronavirus NL63 (HCoV-NL63)
β-CoVs	Bat coronavirus (BCoV) Porcine hemagglutinating encephalomyelitis virus (HEV) Murine hepatitis virus (MHV) Human coronavirus 4408 (HCoV-4408) Human coronavirus OC43 (HCoV-OC43) Human coronavirus HKU1 (HCoV-HKU1) Severe acute respiratory syndrome coronavirus (SARS-CoV) Middle Eastern respiratory syndrome coronavirus (MERS-CoV)
γ-CoVs	Avian infectious bronchitis virus (IBV) Turkey coronavirus (TCoV)

## Origin and Epidemiology

On the New Year's eve of 2020, China notified the World Health Organization (WHO) in a high-priority report regarding the outbreak of new viral infectious disease, and promptly, Wuhan's seafood wholesale market was closed. However, the first case of COVID-19 was diagnosed long back in November 2019. 7 In 7 days after preliminary genomic analysis, the virus confirmed as a coronavirus with more than 95% similarity with the coronavirus of the bat and more than 70% homology with the SARS coronavirus. 8 The first “official” mortality by this virus was reported by the January 9, 2020 by the Chinese government. 9 Because of the Chinese new year, huge crowed back to their homeland and this stimulates and intensified the endemic. Cases in other countries like Japan, South Korea, Iran, and even other provinces of China reported more rapidly than expected. In different countries, airports are equipped with screening machines with a thermometer to detect other signs of symptomatic passengers coming from any other countries which are COVID-19 hotspot and if positive, isolate and test them for COVID-19. Counties like Qatar where Qatar airways play a key role in its economy, resume its flights only for Qatari national and residents. 10 It is vital to mention that while the number tends to zero in well-controlled countries like South Korea, Japan, Taiwan, Germany, Spain, and Italy, but it exponentially increasing especially in Iran, Brazil, Russia, and India, as because these countries underestimate the virus. Now, we know that all people at any age and sex are susceptible to this virus. Infection is mainly transmitted by air via droplets coming from a cough or sneeze by a confirmed or symptomatic individual, but it might have happened from an asymptomatic person before symptoms onset. Symptomatic and asymptomatic patients might transmit the disease as long as signs and symptoms last. 11 The disease can be transmitted mainly by inhalation of air droplets or chiefly by contaminated steel surfaces in bus, subway, lift keypads, or door handles and afterward touching eyes, nose, and mouth. The COVID-19 can also be seen in stool and swage and contaminate the water supply system. 12

## Molecular and Clinical Characteristic

### Molecular Structure

The protein produced by coronavirus is a short and ranging from 76 to 109 amino acids and in regards to size, ranging between 8.4 and 12 kDa. Both protein structures (primary and secondary) elucidate that E has hydrophilic amino terminus including between 7 and 12 amino acids, and is short which is followed by 25 amino acids chain and a huge hydrophobic transmembrane domain (TMD) which is ended by a lengthy hydrophilic carboxyl terminus. This region of TMD consists of a minimum of an amphipathic α-helix which oligomerizes to produce a pore in the membrane. 13

### Notable Features of the SARS-CoV-2 Genome

The comparison of two types of COVID-19 (α and β) shows following two novel features in the genome of SARS CoV-2: (1) with regard to structure and biochemical analysis, SARS-CoV-2 looks to be suitable to adhere to human angiotensin-converting enzyme 2 (ACE2) receptor; and (2) the spike protein of above protein has active cleavage site at S1–2 by insertion of 12 nucleotides where additionally guide to the addition of three O-linked glycans on site's surroundings. 14

### Mutations

This virus can infect humans by binding to ACE2 protein. The mutations that happen in the receptor-binding domain of the virus chiefly influence the binding and subsequently affects the severity of the disease in human. In a very latest report by Allison J. Greaney et al, successfully mapped over 4,000 mutations in SARS-Cov-2 which confirmed their role in the binding ability to ACE2 protein. They successfully identify constrained surfaces as ideal targets for vaccines and antibody therapeutics. 15 On the other hand, in a significant finding, Young et al reported a new and yet effective mutation that can alter the severity of the disease. They stated that COVID-19 variant ∆382 linked to mild COVID-19 infection. The samples of clinical cases suggesting that mutations (mostly deletion) in ORF8 might affect the future of vaccine development and drug discovery and treatments. 16

The very dreadful, the latest mutation, which is called “VUI-202012/01,” consists of a novel mutation in the coronavirus genome which encodes for the spike protein. The outcome of this mutation is much rapid spreading of the COVID-19 based on the first report on this matter which is published by Wise on the *BMJ Journal* . 17 The contagiosity of the above variant is 56% higher than common coronavirus which is reported by The Centre for the Mathematical Modelling of Infectious Diseases in London. 18

### Palmitoylation

The tasks like palmitoylations within proteins and among sections of the membrane shall regulate the protein–protein interactions (PPI). Palmitoylated proteins showing an elevated hydrophobicity that is also observed to help in membrane connection and also active in membrane anchoring. Viral palmitoylated protein is will performed in the virus's envelope such as HA (hemagglutinin protein) of the influenza virus. 19

### Myristoylation

Myristic acid interdependence to the glycine residue's N-terminal is reported in some cellular, bacterial, or viral proteins which is N-terminal myristoylation. Proteins of virus origin have been myristoylated encompass the gag protein of simian immunodeficiency virus (SIV), VP4 protein of poliovirus, HBV's pre-S1 protein (hepatitis B virus), and Nef protein (negative regulatory factor) of HIV (human immunodeficiency virus). 20

### Ubiquitination

Ubiquitinylation and its peer deubiquitination are precise modifications in the posttranslational stage with the role in homeostasis maintenance via adjustment of cellular protein ranges and their activity. COVID-19 can utilize this machinery in the host cell or in extreme cases, encode its enzymes to direct its viral life cycle. 21

### Glycosylation

Inside the N-linked glycosylation, moieties oligosaccharide is adhering to a particular asparagine residue placed in Asn-X-Ser/Thr canonical sequence. This helps for exact enfolding and carrying viral proteins by industriously using host's chaperon proteins including calreticulin and calnexin. 22

## Immune Response: Inflammasome Activation

Viruses including COVID-19 usually encode proteins which meddling with the host defense mechanism to suppress or enhance a response as the basis of their pathogenicity. A couple of these virus-based products interrupt parts of protection system pathways in course of defense to express their version. 23 On the other hand, these proteins shall regulate the host's other cellular mechanisms that might additionally interrupt the immune responses to advance their pathogenicity. Proteins like 2B from coxsackievirus encourage internalization of major histocompatibility complex, class I (MHC-I) protein and meanwhile inhibit their transfer to the surface of the cell as recognition side. 24

## Coronavirus Replication and Pathogenesis

ACE2 which can be seen in the human's lower respiratory tract also works as a receptor for SARS-CoV-2 and additionally modulates both human–human and cross-species transmission. This virus for the very first time in the current outbreak isolated from BALF (bronchoalveolar lavage fluid) of a COVID-19 infected patient. 25 The analysis attested that the virus uses the very same ACE2 receptor. S-glycoprotein on the exterior part of the envelope of coronavirus shall bind to the ACE2 receptor on the surface of human cells. Moreover, S-glycoproteins have two subunits, namely, S1and S2. Receptor-bind domain (RBD) is the key role of S1 and S2 helps membrane fusion of cell and virus-mediated by HR1 and HR2 (heptad repeats 1 and 2). After the fusion of membrane, the RNA of virus freed into the host cell's cytoplasm and the naked RNA translates PP1a and PP1ab which are polyproteins. 26 Their role includes to encode nonstructural proteins and also to form replication transcription (RTX) complex.

## Clinical Features

Pyrexia, pertussis, dyspnea, myalgia, fatigue, pharyngitis, cephalgia, diarrhea, anosmia, and ageusia also reported. 7

## Advanced Drug Therapy Potential as a Powerful Tool in the Near Future

The gold standard for home isolation is to keep yourself hydrated and maintain balanced nutrition to control the fever and cough. Conventional therapies include antibiotic and some antiviral drugs, such as oseltamivir, to be avoided in COVID-19-infected patients. Antifungals and antibiotics are advised to be prescribed if coinfection is confirmed. As of now, there are no approved cure and treatment options for this novel coronavirus. 7

On the other hand, cutting-edge technology is also considered a powerful yet precise and safe option in the war against COVID-19. Khodavirdipour et al in their very latest comprehensive study reported the great achievement by an Israeli firm to develop a cell therapy breakthrough to treat COVID-19 which will be heading for human clinical in a couple of months. 7 Plasma therapy is permitted for investigational purposes by the Food and Drug Administration (FDA) on May 1, 2020. 21 A team from Karolinska Institute, Sweden, developed a new drug to combat COVID-19 which significantly reduces ventilation time for intensive care unit (ICU) admitted patients. 27 clustered regularly interspaced short palindromic repeats (CRISPR)/Cas13 also passing its later stages of treatment option that also tried in many cases as a diagnostic tool.

## Prevention

As mentioned above, as of now, there is no confirmed treatment. Currently, some drugs are under clinical trials for the COVID-19; but till then, prevention is more critical. Numerous characteristics of COVID-19 make prevention uneasy including incubation period, the onset of symptoms, nonspecific properties of the virus, infectivity of COVID-19, illness duration, and more importantly transmission by the recovered patient. Isolation of patients (either suspect or confirmed) with mild illness is highly advised. 28 The house ventilation should be proper and receive enough sunlight during the day for the virus destruction. An individual with a positive and confirmed case has to be asked to wear even a simple surgical mask and practice hygienic during sneezing and coughing. Health care providers also must wear at least a surgical mask when staying in the same room with a COVID-19-infected patient. Washing hands are highly advised for everybody for at least 25 to 30 seconds. 29

## Practice Points from an Iranian Point of View

COVID-19 Iranian story is unique in terms of facts and claims which can ring the bell for a nation-wide disaster. 30 Iran and Sweden are famous failed examples of “herd immunity,” 31 32 but it becomes extremely costly for Iran as the population is eight times more than Sweden. For the safety of our compatriot and other people around the world, following recommendations are advised:

- Health care workers must take down patient's travel history (either within Iran or abroad) with symptoms of shortness of breath and cough, as well as name and contact details of persons who were in touch with the patient. And all of them should be monitored for COVID-19.
- Hospitals and clinics have to set up a separate triage for a patient with COVID-19-positive reports and ask them to wear a mask all the time. And doctors and nurses are strictly advised to wear masks all the time while in contaminated aria/hospital or clinic.
- As soon as possible the case got confirmation of COVID-19, one should immediately be transferred to a referral government-designated hospital or location for further testing and treatment.
- Patients with severe acute respiratory distress syndrome (ARDS) and pneumonia must be checked for travel history and asked to go for the COVID-19 examination.
- Regular disinfection of possible contaminated areas and surfaces should be disinfected by 70% alcohol.
- All doctors and medical staff advised keeping themselves updated by the latest findings, treatment, and prevention options.
- All unnecessary travels inside or outside of the hometown should be banned.
- People must kindly stop assume in myths and put an end to spreading superstitious believes and try to calm family ad relatives by phone or voice calls and stop visiting them in person.

## Vaccine Development Update

In a very latest update by January 2021, overall 165 vaccines registered for trials and 30 vaccines already entered the human trial phases. In this current update, five new entries were observed including ReiThera from Italy, Medigen from Taiwan, and Zydus-Cadila's Indian subsidiary all in phase-I and Novavax at the preliminary stage with promising results. As for public knowledge, vaccine development including several phases such as preclinical stage, phase-I which is safety trials, phase-II which is expanded trials, phase-III which is efficacy trials, and finally heading for approval if passed all phases. But due to extreme pressure on biotechnology and pharmaceutical companies, they decided to go for a “combined phase,” in which phases I and II have been combined. There are three vaccines in the market right now and approval by FDA/EMA (European Medicines Agency) including Moderna's mRNA-based vaccine to produce viral protein, BioNtech-Pfizer joint venture vaccine and Oxford-AstraZeneca's ChAdOx1. 33

## Approved Vaccines and New Mutation

“World's first approved vaccine from Pfizer-BioNtech will withstand new mutation without any worry to lose its efficacy,” as said by Philip Dormitzer, Pfizer's Vice President and Chief Scientific Officer of viral vaccines. 34

## Conclusion

The outbreak of COVID-19 challenged public health, medical services, and the economic infrastructure of the world. As of now, only “time” will tell us how this outbreak will change and reshape our life in the future. This review tried to cover a concise update on the genomics of SARS-CoV-2 and its immunopathogenesis and infection, along with a brief comparison of conventional therapies versus latest trials on cell therapy and CRISPR/Case13. The relation between the severity of diseases in different individuals with their immune system dysfunctionality stated. These factors should be considered in the procedures of vaccine development. More research is needed to evaluate the efficacy of offered treatment options and also more molecular studies to a deeper knowledge of the virus itself. This may help in finding the specific biomarkers in the near future.
